# Prediction of fatigue life of high-heat-load components made of oxygen-free copper by comparing with Glidcop

**DOI:** 10.1107/S0909049512041192

**Published:** 2012-11-10

**Authors:** Sunao Takahashi, Mutsumi Sano, Atsuo Watanabe, Hideo Kitamura

**Affiliations:** a JASRI, SPring-8, 1-1-1 Kouto, Sayo-cho, Sayo-gun, Hyogo 679-5198, Japan; b RIKEN SPring-8 Center, 1-1-1 Kouto, Sayo-cho, Sayo-gun, Hyogo 679-5148, Japan

**Keywords:** OFC, front ends, high heat load, Glidcop, thermal limitation, strain-range partitioning method, creep fatigue

## Abstract

Following a successful study on the prediction of fatigue life of high-heat-load components made of Glidcop, the thermal limitation of oxygen-free copper (OFC), which is used more commonly than Glidcop, has been studied. In addition to its general mechanical properties, the low-cycle-fatigue (LCF) and creep properties of OFC were investigated in detail and compared with those of Glidcop. The breaking mode of OFC, which was observed to be completely different from that of Glidcop in a fatigue fracture experiment, clarified the importance of considering the creep–fatigue interaction. An additional LCF test with compressive strain holding was conducted so that the creep–fatigue life diagram for out-of-phase thermal fatigue could be obtained on the basis of the strain-range partitioning method. The life predicted from elasto-plastic creep analysis agreed well with that determined from the void ratio estimated in the fatigue fracture experiment.

## Abstract 2

By using the strain-range partitioning method, the fatigue life of high-heat-load components made of oxygen-free copper have been successfully predicted within a factor of two.

## 1. Introduction   

Oxygen-free copper (OFC), a high-purity copper free of oxides, is one of the most popular materials used in many accelerator facilities around the world. In particular, OFC is typically used as a heat-load-absorbing material in vacuum owing to its high thermal conductivity and low outgassing characteristics. On the other hand, Glidcop, a dispersion-strengthened copper with aluminium oxide ceramic particles, has been applied to high-heat-load components instead of OFC owing to its extremely high heat resistance in the third-generation synchrotron radiation facilities such as SPring-8 (Mochizuki *et al.*, 2004 ▶; Oura *et al.*, 1998 ▶). It is well known that the high-temperature properties of Glidcop are far superior to those of OFC (Miller *et al.*, 1991 ▶). Furthermore, a previous paper (Robles *et al.*, 1994 ▶) reported that the main purpose of adding alumina particles to copper is to not only impart strength but also prevent macro-crack formation, because these particles act as obstacles in the path of a propagating crack field. In a previous study we investigated the thermal and mechanical properties of Glidcop and successfully established a procedure to predict the fatigue life of high-heat-load components made of Glidcop to within a factor of two by consolidating experimental and analytical results (Takahashi *et al.*, 2008 ▶). However, from an economic point of view, application of Glidcop to all irradiated components is not a sensible option. Therefore, the aim of this study was to establish guidelines for selecting either OFC or Glidcop, as per the requirement.

## 2. Mechanical properties of OFC as compared with Glidcop   

We conducted tension, low-cycle-fatigue (LCF) and creep tests on OFC to delineate the differences between OFC and Glidcop. As the properties of OFC depend in a large part on the grade (purity) and thermal treatment, the tested OFC was chosen to be identical to that used at the SPring-8 front end, namely C1011BE (specified in JIS and equivalent to C10100-M30 in ASTM), with as-manufactured temper. The reference material, Glidcop AL15, was heat-treated in advance so that its thermal history during brazing in an actual manufacturing process might be considered (Takahashi *et al.*, 2008 ▶).

### 2.1. General properties   

**Figure 1:**
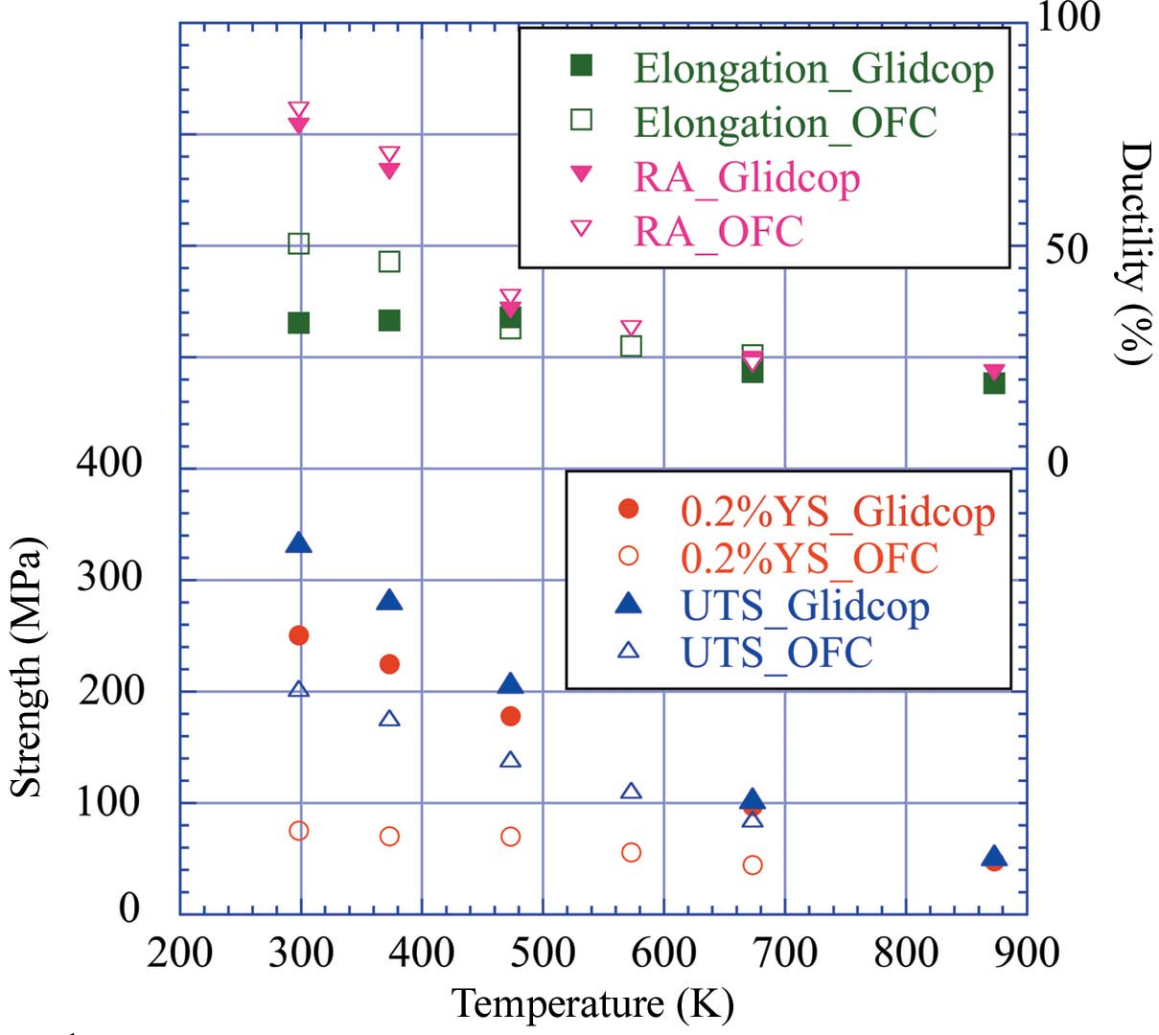
Results of a tension test on OFC along with the data on Glidcop for comparison.

A general tension test was carried out at temperatures of 293, 373, 473, 573, 673 and 873 K. Fig. 1 ▶ shows the test results, namely, the ultimate tensile strength, 0.2% yield strength, elongation, and reduction in area, along with the corresponding data for Glidcop. As a general trend, Glidcop was superior to OFC in terms of strength whereas OFC was superior to Glidcop in terms of ductility at temperatures below 373 K. It should be noted that, as the yield strength of OFC is extremely small even at room temperature, plastic deformation could occur easily.

### 2.2. Fatigue properties   

#### 2.2.1. LCF test in vacuum   

**Figure 2:**
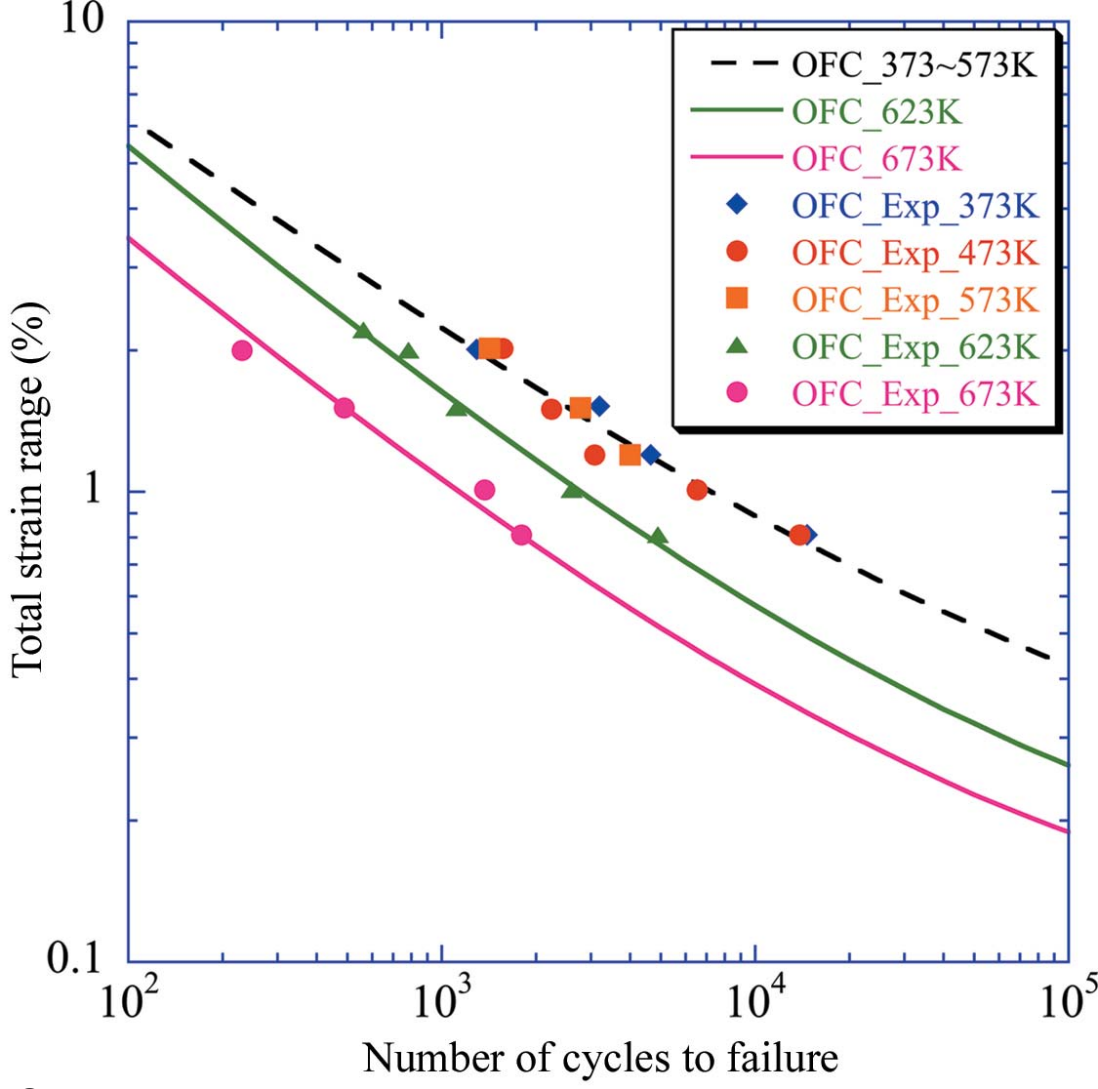
Results of a LCF test in vacuum (markers) and the relationship between Δ∊_t_ and *N*_f_ derived from formula (dashed line for the range 373–573 K and solid lines for 623 and 673 K).

Fig. 2 ▶ shows the results of a strain-controlled LCF test in vacuum for a total strain range of 0.8% to 2.0% at temperatures of 373, 473, 573, 623 and 673 K. We defined the fatigue life as the number of cycles at which the peak tensile stress during each cycle reduced by 25% from its initial value (JIS Z2279:1992). Differences among the data at 373, 473 and 573 K were so small that a single fatigue life diagram sufficed in the range 373–573 K. Compared with this diagram, the data at 673 K showed a significant reduction. Interestingly, the data at 623 K were located at nearly intermediate points between those at 373–573 K and at 673 K. This suggests that the LCF property of OFC will decrease continuously when the temperature is 573 K or greater.

We analysed the data based on the Manson–Coffin equation, which is given below, by dividing the temperature range into two regions: 373–573 K and 573–673 K,
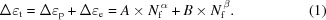
Here, Δ∊_t_, Δ∊_p_ and Δ∊_e_ are the total, plastic and elastic strain ranges, respectively; *A*, *B*, α and β are the material properties shown in Table 1 ▶; and *N*_f_ is the number of cycles to failure. The coefficients *A* and *B* in the range between 573 and 673 K are expressed as a function of temperature (K). The relationship between Δ∊_t_ and *N*_f_ derived from equation (1) is also shown in Fig. 2 ▶.

#### 2.2.2. Comparison with Glidcop   

**Figure 3:**
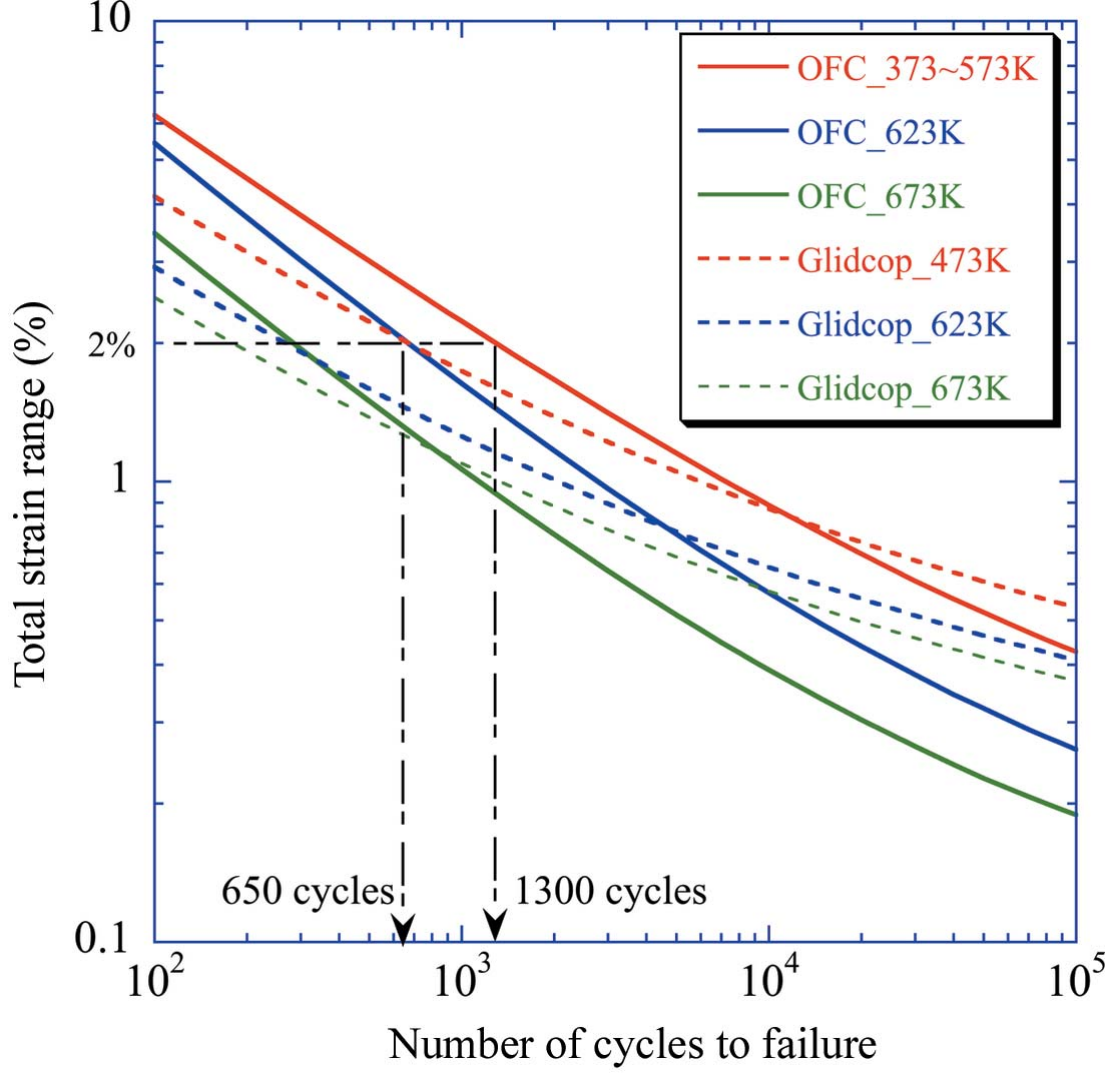
Comparison of the fatigue property between OFC and Glidcop (solid lines for OFC and dashed lines for Glidcop).

Fig. 3 ▶ shows a comparison of the LCF property in vacuum between OFC and Glidcop. At temperatures below 573 K the LCF property of OFC is better than that of Glidcop in the higher-strain-range region. For example, when Δ∊_t_ is 2% at a temperature of 473 K, *N*_f_ for OFC (1300) is twice as long as that for Glidcop (650). The fatigue properties of both materials become equal at a Δ∊_t_ of 0.8%, in which case *N*_f_ is about 10^4^. As the temperature increases, the intersection moves towards the lower side of *N*_f_. Consequently, we conclude that OFC is superior to Glidcop in the LCF region, whereas Glidcop is superior to OFC in the high-cycle fatigue region. This tendency becomes pronounced as the temperature increases.

### 2.3. Creep properties   

#### 2.3.1. Larson–Miller parameter   

**Figure 4:**
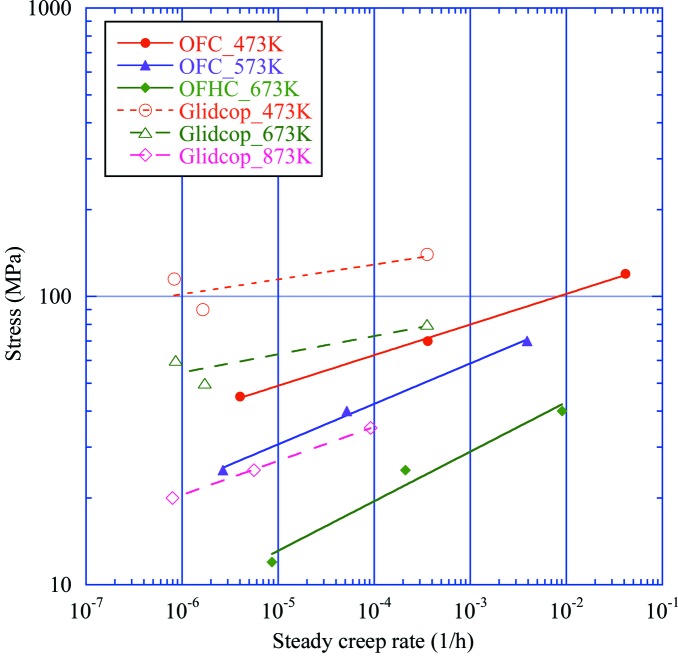
Relationships between stress and steady creep rate at temperatures of 473, 573 and 673 K. Data on Glidcop are also shown (outlined markers) for comparison.

**Figure 5:**
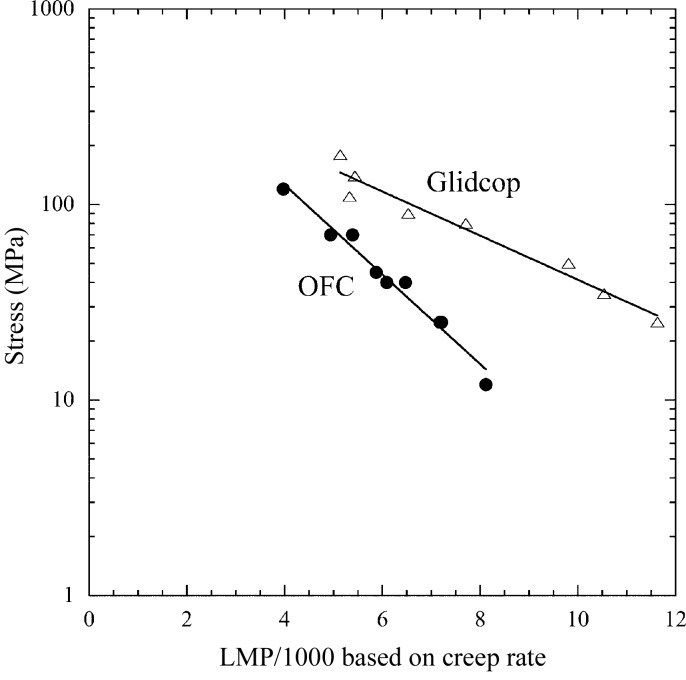
Relationships between stress and Larson–Miller parameter for OFC and Glidcop.

We conducted creep tests for OFC at temperatures of 473, 573 and 673 K. Fig. 4 ▶ shows the relationship between the stress and steady creep rate at each temperature along with the results for Glidcop for comparison. To prepare a creep constitutive equation for finite-element-method (FEM) analysis, we introduced the Larson–Miller parameter (LMP), given by equation (2), which indicates the influence of holding time and temperature on creep damage,
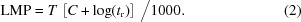
Here, *T* is the holding temperature (K), *t*_r_ is the rapture time (h) and *C* is a material-specific constant, which was determined to be 7 for OFC and 10 for Glidcop by fitting the test results shown in Fig. 4 ▶. As shown in Fig. 5 ▶, for both materials, each set of three creep curves at different temperatures (Fig. 4 ▶) can be arranged into one curve with the LMP.

#### 2.3.2. Comparison with Glidcop   

For example, under a stress of 90 MPa at 473 K, the steady creep rate of 1.5 × 10^−6^ for Glidcop is about three orders of magnitude less than that of 3 × 10^−3^ for OFC. The creep property of Glidcop at 873 K is roughly the same as that of OFC at 573 K. In this sense Glidcop can be regarded as far superior to OFC in creep property regardless of temperature range. This superiority becomes more remarkable as the temperature rises. These data imply that the influence of creep should be considered while selecting OFC.

## 3. Fatigue fracture experiment   

### 3.1. Experimental set-up   

We carried out repeated electron-beam-irradiation experiments with a specimen made of OFC according to the same procedure as that in the case of Glidcop (Takahashi *et al.*, 2008 ▶). Briefly, the test piece consisted of an absorbing body made of OFC and a cooling holder made of stainless steel, both of which were fastened by 12 bolts. It was specially designed with the intention of locally concentrating the strain in the central area of the absorbing body. The experiment was conducted on the sample with a normal incidence angle. One irradiation cycle is comprised of a 7 min thermal loading condition and a 5 min unloading condition.

### 3.2. Experimental results   

**Figure 6:**
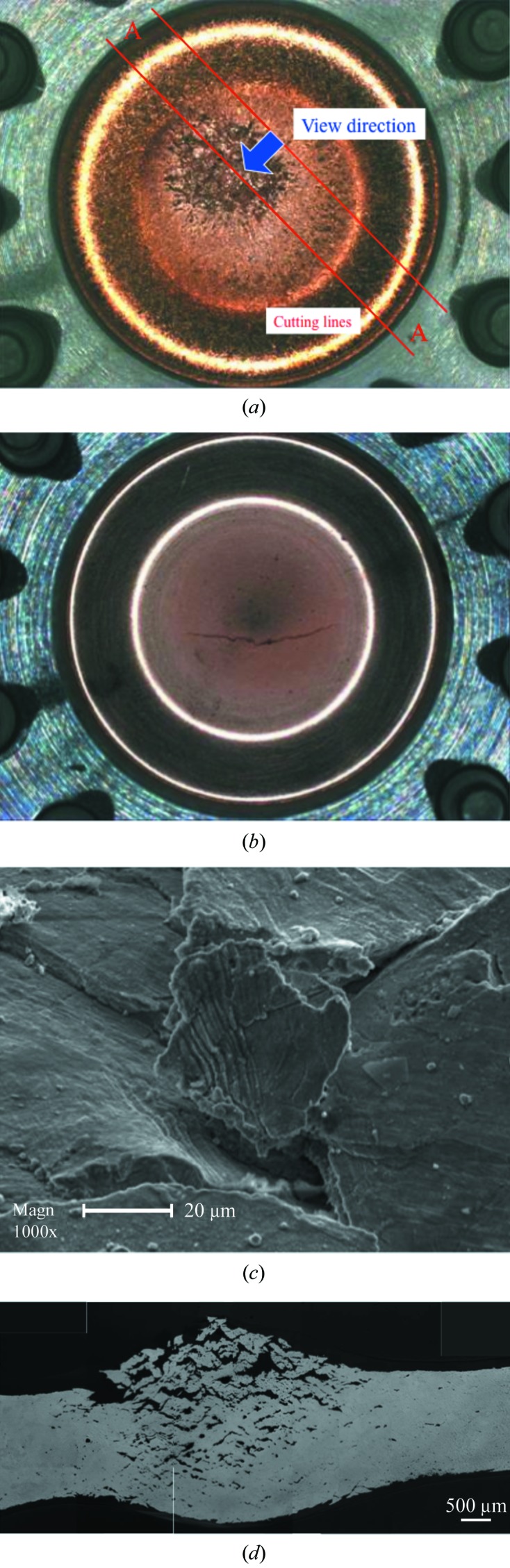
(*a*) Photograph of the overall view of the absorbing surface of the OFC specimen after 1000 cycles with a total power of 550 W. (*b*) Photograph of the overall view of the absorbing surface of Glidcop specimen for comparison after 75 cycles with 650 W, 160 times with 600 W, and 265 cycles with 550 W. (*c*) Field-emission scanning electron microscopy photograph of the area around the centre of (*a*) at 1000× magnification. (*d*) Photograph of micro-structural observation at the *A*–*A* cross section shown in (*a*).

**Figure 7:**
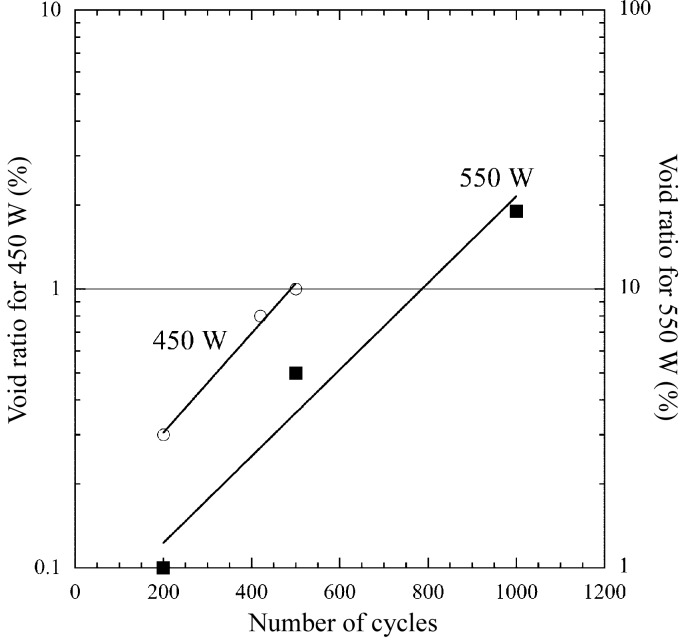
Relationship between void ratio and number of cycles for absorbed powers of 450 and 550 W.

First, we conducted an experiment with an absorbed power of 550 W. In this case the peak power density was 40.7 W mm^−2^, and the resulting maximum temperature was expected to reach about 850 K. Fig. 6(*a*) ▶ shows an overall view of the absorbing surface after 1000 cycles. The corresponding photograph for the case of Glidcop is also shown in Fig. 6(*b*) ▶ for comparison. In the latter case a number of small cracks had emerged, one of which propagated linearly into a macro-crack fracture. We assume that this is because alumina particles, the admixture of Glidcop, do not dissolve in copper, and therefore the interface between copper and aluminium becomes the origin of stress concentration. In contrast, although a large number of micro-cracks heavily populate OFC, they never become interconnected as they do in Glidcop. As shown in Fig. 6(*c*) ▶, which is a magnification of Fig. 6(*a*) ▶, each crack is apparently a kind of intergranular fracture. This cannot be regarded as a fracture if we use the same evaluation method as that used for Glidcop (*i.e.* one based on the propagated macro-crack length) to determine the observed fracture life of OFC. However, micro-structural observations at the *A*–*A* cross section shown in Fig. 6(*a*) ▶ reveal that voids and cracks are present from the surface to the back of the sample (Fig. 6*d* ▶). These defects were concentrated at grain boundaries, which presumably occur owing to the initiation and coalescence of grain boundary cavities caused by creep at elevated temperatures. This implies that the fracture phenomenon in OFC is significantly influenced by the fatigue–creep inter­action. Volume expansion was also observed in the absorbing section. To all appearances this condition should cause failure of proper OFC function in terms of vacuum and cooling water leakage issues. Therefore, we introduced another evaluation method based on a void ratio. We performed image binarization by digital image processing on a cross-sectional photograph. The target domain for image binarization was limited to a circular region equivalent to the FWHM of the electron beam. We defined the void ratio as the area of voids in a mixture divided by the total area (solid + void), and estimated the void ratio after 1000 cycles to be 19%. We conducted additional experiments with 200 and 500 cycles for 550 W as well as 200, 420 and 500 cycles for 450 W, and then measured the void ratios in each case. Fig. 7 ▶ shows the relationship between the number of cycles and the void ratio.

## 4. Prediction of fatigue life   

### 4.1. Strain-range partitioning method   

To estimate the cumulative damage under the creep–fatigue interaction, we applied the strain-range partitioning method (Manson, 1973 ▶), which is one of the most powerful damage rules. In this method inelastic strain ranges are partitioned into four distinct fundamental components according to the deformation direction (tensile or compressive) and time-dependency (creep or plastic): (i) type pp (completely reversed plasticity; Δ∊_pp_), (ii) type pc (tensile plasticity reversed by compressive creep; Δ∊_pc_), (iii) type cp (tensile creep reversed by compressive plasticity; Δ∊_cp_), and (iv) type cc (completely reversed creep; Δ∊_cc_). The LCF test mentioned earlier in §2.2 is used to assess Δ∊_pp_. Each of these fundamental components is considered to obey the Manson–Coffin law independently. Any repeated inelastic strains can be expressed by combining the individual components, thus enabling the prediction of fatigue life.

### 4.2. LCF tests with compressive/tensile strain holding   

**Figure 8:**
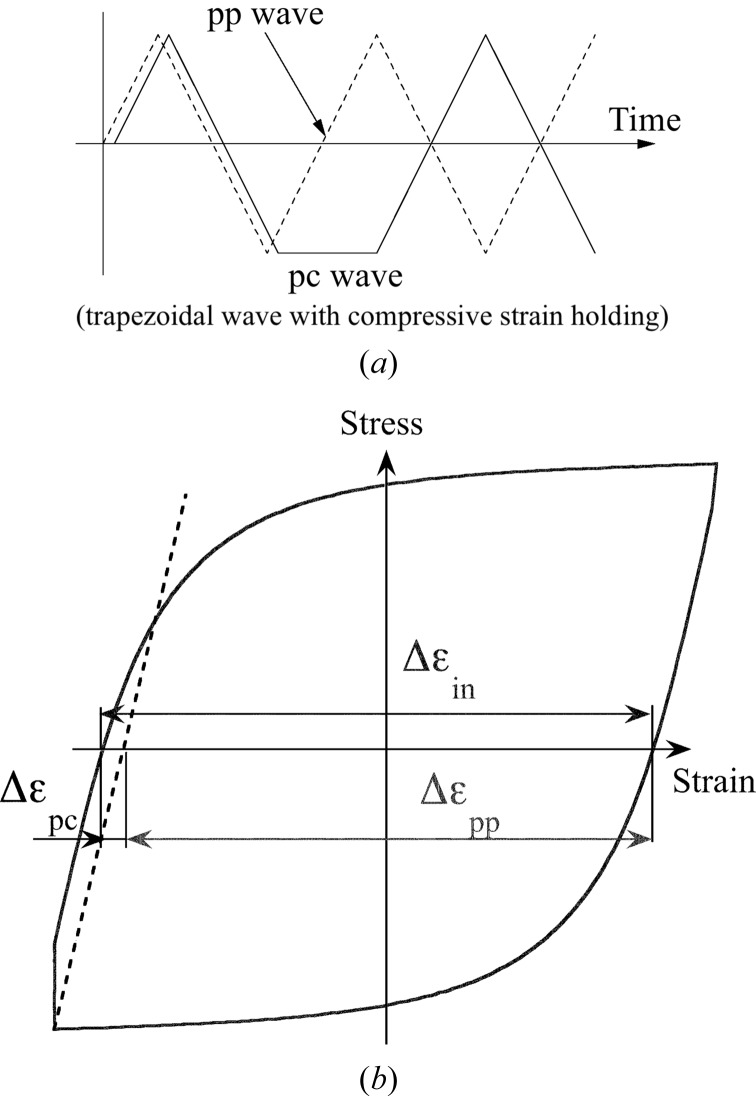
(*a*) Wave forms at the LCF test with compressive strain holding (trapezoidal pc wave; solid line) and without strain holding (triangular pp wave; dashed line). (*b*) Partitioning of the inelastic strain range in a closed stress–strain hysteresis loop for the pc wave.

**Figure 9:**
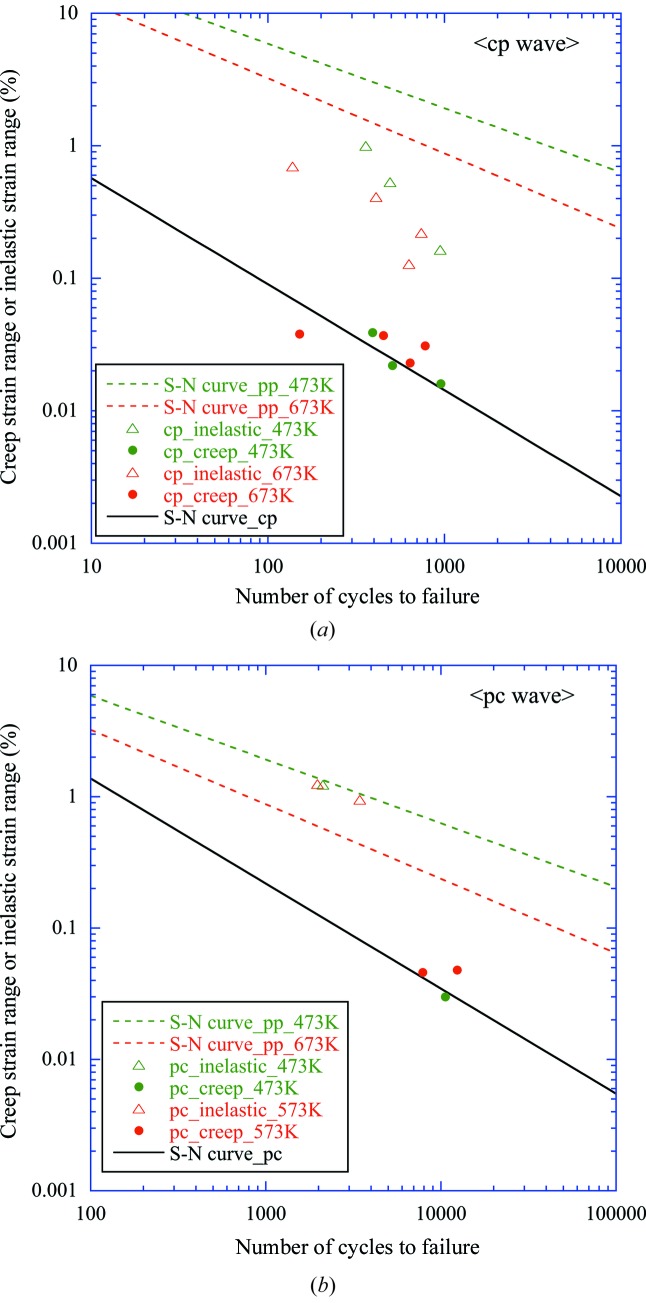
LCF test results (open symbols) and resulting relationship between *N*_*ij*_ and Δ∊_*ij*_ (solid symbols) for cp (*a*) and pc waves (*b*), respectively. The *N*_pp_–Δ∊_pp_ relationship (dashed lines) and the final obtained creep–fatigue life diagram (black solid line) are also shown for each case.

**Figure 10:**
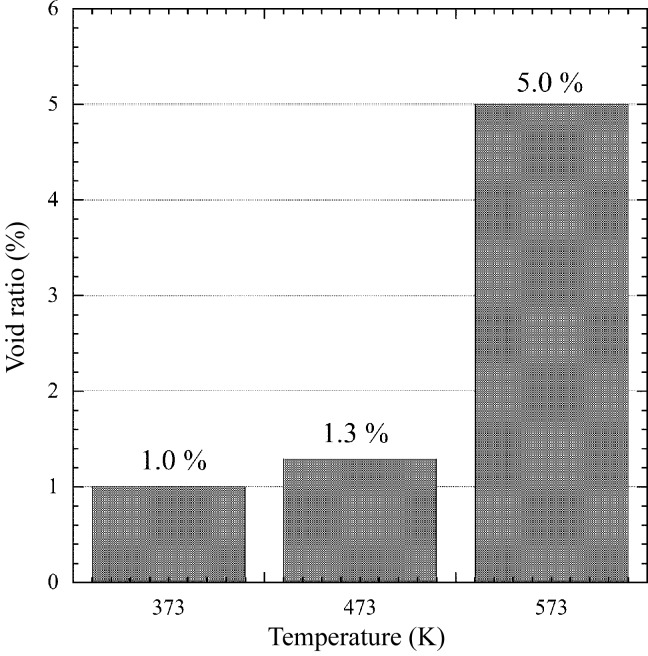
Void ratio of specimen at number of cycles to failure in the LCF test with the pc wave at temperatures of 373, 473 and 573 K. The void ratio at 573 K increases rapidly.

Additional LCF tests were conducted in vacuum with both tensile and compressive strain holding for the total strain range of 0.2% to 1.2% at temperatures between 373 and 673 K. The specific procedures to obtain a creep–fatigue life diagram, *i.e.* the relationship between Δ∊_*ij*_ and *N*_*ij*_ (*ij* = cp or pc), in reference to the case of compressive strain holding are as follows: (i) measure the number of cycles to failure (*N*_f_) for a trapezoidal pc wave pattern shown in Fig. 8(*a*) ▶; (ii) partition the inelastic strain range in the closed stress–strain hysteresis loop at a cycle of about *N*_f_/2 into Δ∊_pp_ and Δ∊_pc_ (Fig. 8*b* ▶); (iii) calculate *N*_pp_, which corresponds to Δ∊_pp_, according to equation (1); and (iv) estimate *N*_pc_, which corresponds to Δ∊_pc_, by using the linear cumulative fatigue damage rule, as shown in equation (3), to derive the *N*_pc_–Δ∊_pc_ diagram,
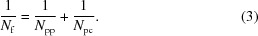
Figs. 9(*a*) and 9(*b*) ▶ show the LCF test results (open markers) and the resulting relationship between *N*_*ij*_ and Δ∊_*ij*_ (solid markers) for the cp wave and pc wave, respectively. The *N*_pp_–Δ∊_pp_ relationship (dashed lines) at temperatures of 473 and 673 K are also shown for reference. We concluded that the temperature dependency of the test results at the test temperatures was so small in each case that all of the data could be consolidated. Thus a single diagram was independently developed for the pc wave and cp waves, as expressed in equations (4) and (5), respectively. The slope of the creep–fatigue diagram was set at −0.8, which is why this value has been commonly employed based on Manson’s study on a wide variety of materials (Manson, 1973 ▶). Our cp wave test results showed fairly good agreement with this concept,
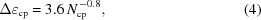


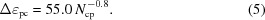
Because the type of thermal fatigue for the fatigue fracture experiment is out of phase (compressive at high temperature and tensile at low temperature), we measured the void ratio of the specimens at the number of cycles to failure for the test with compressive strain holding (pc wave) at temperatures of 373, 473 and 573 K. As shown in Fig. 10 ▶, the void ratio is fairly small at temperatures below 473 K (about 1%), but increases to 5% at 573 K. These values are used as criteria to determine the observed fatigue life in the fatigue fracture experiment.

### 4.3. FEM analysis   

**Figure 11:**
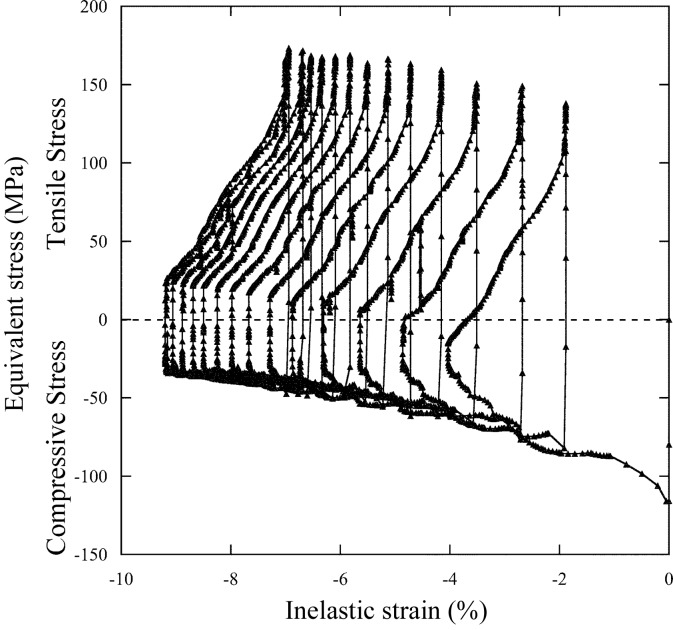
Hysteresis loop of the inelastic strain and equivalent stress based on elasto-plastic creep analysis.

**Figure 12:**
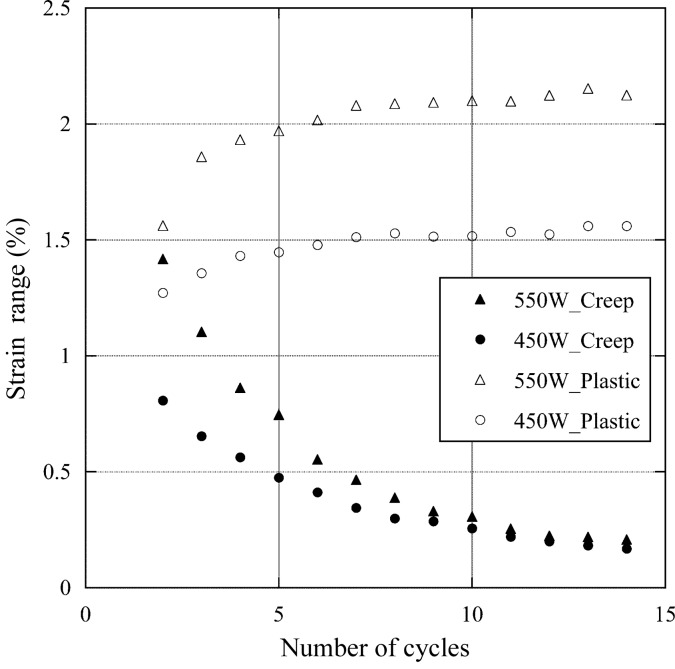
Relationships between number of cycles and creep strain range (solid symbols) as well as plastic strain range (open symbols).

Along with the fatigue fracture experiment, we performed elasto-plastic creep analysis by employing the finite-element analysis program *ANSYS* (http://www.ansys.com/). The modeling and boundary conditions for the analysis followed the case of Glidcop (Takahashi *et al.*, 2008 ▶), and only the physical properties were modified. We applied the stress–strain property obtained at the LCF rather than the tension test, considering the effect of softening or hardening of the material caused by cyclic load at elevated temperatures. The Norton model was selected for setting the creep constitutive equation. A hysteresis loop of the inelastic strain and the equivalent stress, shown in Fig. 11 ▶, was drawn by the elemental solutions of the center element in the absorbing surface when a cyclic heat load of 550 W was applied 14 times. The relationships between the cyclic number and the plastic strain range as well as the creep strain range are shown in Fig. 12 ▶, including the case of 450 W. After 14 heat cycles, the inelastic strain range converges to about 2.3% and is composed of 2.1% plastic strain range (Δ∊_pp_) and 0.2% creep strain range (Δ∊_pc_) for 550 W.

### 4.4. Discussion   

**Figure 13:**
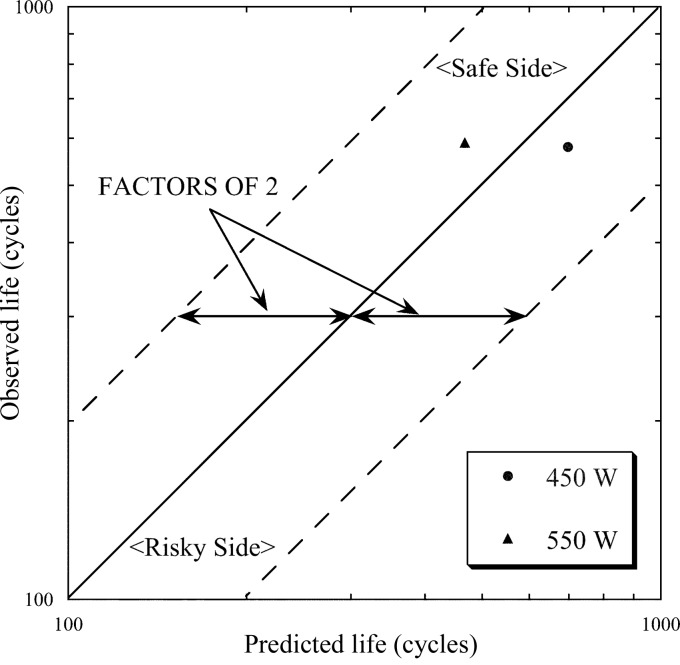
Relationship between observed and predicted fatigue life. The two dashed lines indicate life differing by a factor of two.

Here we discuss the creep–fatigue life on the basis of the strain-range partitioning method, using the above-mentioned results. In the case of 550 W, *N*_pp_ and *N*_pc_, corresponding to 2.1% Δ∊_pp_ and 0.2% Δ∊_pc_, respectively, were calculated to be 837 cycles and 1120 cycles according to equations (1) and (5), respectively. Consequently, the predicted fatigue life derived from equation (3) was estimated to be about 467 cycles. All calculated strain ranges and fatigue lives are shown in Table 2 ▶. On the other hand, the value of the void ratio for a criterion of the observed life of the fatigue fracture experiment (Fig. 7 ▶) was determined based on the results of Fig. 10 ▶ at an arithmetic mean temperature with a high temperature corresponding to the maximum and a low temperature of 303 K. As shown in Table 2 ▶, as the mean temperature was 572 and 496 K for the absorbed powers of 550 and 450 W, the criteria were set according to Fig. 10 ▶ to be 5% (void ratio at 573 K) and 1.3% (void ratio at 473 K), respectively. Consequently, we regarded the observed lives for 550 and 450 W to be 590 and 553 cycles, respectively. Fig. 13 ▶ shows the relationship between the predicted and the observed life. The two dashed lines indicate lives differing by a factor of two. They can be said to be in extremely good agreement.

## 5. Conclusions   

Several mechanical property tests were conducted on OFC to delineate the differences between OFC and Glidcop, and we confirmed that (i) plastic deformation of OFC could easily occur because its yield strength is extremely small at room temperature; (ii) OFC is superior to Glidcop in the low-cycle fatigue region at temperatures below 573 K whereas Glidcop is superior to OFC in the high-cycle fatigue region, and this tendency becomes pronounced as the temperature increases; and (iii) Glidcop is far superior to OFC in creep properties regardless of the temperature range, and this superiority becomes more marked as the temperature rises.

We applied a cyclic heat load to a specially designed specimen made of OFC by using an electron beam irradiation system and measured the void ratio of the specimens to determine the observed fatigue life. The fracture behavior of OFC, which was completely different from that of Glidcop, indicated that the fracture morphology of OFC is apparently dominated by cavity-type grain-boundary cracking. This suggests that a fatigue–creep interaction should be considered to deal with the fracture phenomenon of OFC. Accordingly, we conducted another LCF test in vacuum with compressive strain holding to obtain a creep–fatigue life diagram (Δ∊_pc_–*N*_pc_) by introducing the strain-range partitioning method. We measured the void ratio of the specimens at the number of cycles to failure at each temperature to decide the criteria for the observed fatigue life. The void ratio was fairly small at temperatures below 473 K (∼1%), but it increased to 5% rapidly at 573 K. In addition, elasto-plastic creep analysis was conducted to predict fatigue life on the basis of the strain-range partitioning method. The results confirmed that, by considering the effect of the creep–fatigue interaction, the observed life of OFC was within a factor of two of the predicted life.

We wish to note the following suggestions about the usage of OFC and Glidcop: (i) the effect of creep–fatigue interaction on fatigue life when using OFC at elevated temperatures should be considered; (ii) OFC should not be used under plastic deformation at temperatures above 573 K (473 K is a safer temperature), according to Fig. 10 ▶; and (iii) differences in the breaking mode of both materials should be taken into consideration.

## Figures and Tables

**Table 1:** Material properties in vacuum of *A*, *B*, α and β in equation (1) *A* and *B* are constant in the range 373–573 K but vary independently as a function of temperature (K) in the range 573–673 K.

Temperature	Manson–Coffin		Basquin	
*T* (K)	*A*	α	*B*	β
373–573	55.3	−0.486	0.49	−0.069
623	70.0	−0.567	0.45	−0.090
673	44.0	−0.567	0.35	−0.090
573–673	1390 exp[−8.60 × 10^−3^(*T* − 273)]	−0.567	2.15 exp[−4.52 × 10^−3^(*T* − 273)]	−0.090

**Table 2:** Analysis results, including boundary conditions, for heat loads of 550 and 450 W The mean temperature, with a high temperature corresponding to the maximum and a low temperature of 303 K. Various estimated lives according to equation (1) for *N*_pp_, equation (5) for *N*_pc_ and equation (3) for *N*_f_ are also listed.

Total power	Maximum power	Body temperature (K)		Strain range (%)		Estimated life (number of cycles)		
(W)	density (W mm^−2^)	Maximum	Mean	Δ∊_pp_	Δ∊_pc_	*N*_pp_	*N*_pc_	*N*_f_
550	40.7	841.7	572	2.1	0.21	837	1054	467
450	24.0	728.1	496	1.5	0.19	1673	1194	697
